# Liver sinusoidal endothelial cells induce tolerance of autoreactive CD4^+^ recent thymic emigrants

**DOI:** 10.1038/srep19861

**Published:** 2016-01-22

**Authors:** Xi Xu, Rong Jin, Mingyang Li, Ke Wang, Shusong Zhang, Jie Hao, Xiuyuan Sun, Yu Zhang, Hounan Wu, Jun Zhang, Qing Ge

**Affiliations:** 1 Key Laboratory of Medical Immunology, Ministry of Health. Department of Immunology, School of Basic Medical Sciences, Peking University Health Science Center, 38 Xue Yuan Road, Beijing, 100191, P. R. China; 2 Center for Molecular Metabolism, Nanjing University of Science and Technology, Nanjing 210094, P. R. China; 3 Peking University Medical and Health Analytical Center, Peking University Health Science Center, Beijing, P. R. China

## Abstract

The liver is a unique lymphoid organ whose microenvironment is biased towards tolerance induction. We previously found that a proportion of CD4^+^ autoreactive recent thymic emigrants (RTEs) retained in the liver after thymic egress and acquired IL-10 producing capability. To investigate the tolerance of these liver persisting CD4^+^ RTEs in more detail and to study the liver stromal cell types that facilitate the tolerogenic changes in young T cells, the phenotype and function of liver RTEs were further characterized and the impact of liver sinusoidal endothelial cells (LSECs) and Kupffer cells on RTEs were examined using an *in vitro* co-culture system. More than 70% of CD4^+^ CD44^hi^ RTEs in the liver acquired Foxp3^-^LAG3^+^ CD49b^−^ regulatory phenotype and function. But higher ratio of apoptosis with enhanced FasL and Bim expression was also found in these CD4^+^ liver RTEs when compared to those in the lymph nodes and spleen. LSECs played an important role in RTEs’ acquisition of tolerogenic and regulatory phenotype. These results indicate an important role of liver microenvironment in enforcing peripheral tolerance to CD4^+^ thymic emigrants against self- and gut-derived antigens.

The liver is a unique lymphoid organ whose microenvironment is biased towards tolerance induction. Such tolerance occurs when mature T cells encounter self or foreign antigens in the liver^1^, including administration of foreign antigens via the portal vein or mouth^2^,^3^, allogeneic liver transplantation^4^, and hepatotropic virus infection^5^,^6^. Even in the absence of antigen, the liver is known to selectively sequester and delete CD8^+^ T cells activated at sites distant from the liver^7^,^8^,^9^,^10^. The microenvironment in the liver induces T cell apoptosis and the acquisition of anergic phenotype in CD8^+^ T cells and regulatory functions in CD4^+^ T cells^11^,^12^,^13^,^14^,^15^.

The liver contains many distinct cell subsets that manifest antigen presenting cell (APC) activity with regulatory or tolerogenic features. These include dendritic cells (DCs), Kupffer cells (KCs), sinusoidal endothelial cells (LSECs), hepatic stellate cells, and even hepatocytes. The unique architecture of the hepatic sinusoids allows circulating T cells to make direct contact with these APCs and recognize self-, neo-, and gut-derived antigens presented by them^16^,^17^. The tolerogenic APCs in the liver express inhibitory or immunoregulatory molecules including prostaglandin E_2_ (PGE_2_), PD-L1, Fas ligand (FasL), LSECtin, and IL-10 which down-regulate the numbers and effector functions of antigen-specific T cells^17^,^18^,^19^. In addition, LSECs constitutively express adhesion molecules such as integrin ligands, intercellular adhesion molecules 1 (ICAM-1), and vascular cell adhesion molecule-1 (VCAM-1), facilitating the sequestration of activated T cells, in particular, CD8^+^ T cells^8^.

We previously identified a unique population of CD4^+^ recent thymic emigrants (RTEs) in the liver^20^. When adoptively transferred into lymphopenic RAG1^−/−^ mice, liver-derived RTEs induce more severe inflammatory cell infiltration in the lung, liver, and intestine than lymph node (LN) RTEs, suggesting that liver may retain a proportion of autoreactive CD4^+^ RTEs that just escape from negative selection in the thymus. The adoptive transfer of thymic RTE precursors (mature Qa2^+^ CD4^+^ single positive (SP) thymocytes) into MHC II^−/−^, *Ltbr*^−/−^, or wild type recipients, or direct analysis of GFP^+^ CD4^+^ RTEs from RAG2p-GFP transgenic mice reveal that these liver persisting autoreactive RTEs acquire activated phenotype (CD44^hi^CD62L^lo^PD-1^lo^Foxp3^−^) in a MHC II-dependent and secondary lymphoid organ-independent manner. When compared to RTEs in the lymph nodes, RTEs in the liver produce higher level of IL-10 upon activation and respond poorly to IL-7-induced survival, indicating that these RTEs acquire tolerogenic properties in the liver microenvironment. To investigate the mechanisms of hepatic tolerogenicity to autoreactive young T cells that just emigrate from the thymus, the current study characterized the phenotype and function of CD4^+^ liver RTEs in more details and then studied the impact of liver KCs and LSECs on RTEs’ acquisition of tolerogenic phenotype.

## Methods

### Mice

C57BL/6 congenic mice (CD45.1^+^ and CD45.2^+^) were purchased from Peking University Health Science Center (Beijing, China). FVB-Tg (Rag2-EGFP) 1Mnz/J mice were purchased from Jackson Laboratory (Bar Harbor, ME) and were backcrossed 10 generations onto the C57BL/6 background (termed as RAG2p-GFP in this paper). Mice were used at 5-11 weeks of age unless stated otherwise. The animals were kept in a specific pathogen-free facility at Peking University Health Science Center (Beijing, China). The experimental procedures on use and care of animals had been approved by the ethics committee of Peking University Health Science Center. This study was carried out in accordance with these approved guidelines.

### Reagents

Anti-Fas (15A7), anti-FasL (MFL3), anti-LAG3 (eBioC9B7W), anti-TIGIT (GIGD7) and anti-FoxP3 (FJK-16s) were purchased from eBioscience (San Diego, CA, USA). Anti-Qa-2 (695H1-9-9) and anti-F4/80 (BM8) were purchased from BioLegend (San Diego, CA, USA). Anti-C3 (RmC11H9) was purchased from Cedarlane (Canada). Annexin V and PI were purchased from Biosea (Beijing, China). All other antibodies used in the study were purchased from BD Biosciences (San Diego, CA, USA). Percoll and Ficoll were purchased from Solarbio (Beijing, China) and Huajing Biotechnology (Shanghai, China), respectively. Recombinant mouse IL-7, Flt3 ligand and recombinant human TGF-β1, IL-2 were purchased from R&D Systems (Minneapolis, MN, USA). Collagenase IV was purchased from Invitrogen (Grand Island, NY, USA). DNase was purchased from Roche (Basel, Switzerland). γ-secretase inhibitor (N-[N-(3.5-difluorophenacetyl)-L-alanyl]-S-phenyl glycinet-butyl ester, DAPT) was purchased from Calbiochem (San Diego, CA, USA). Mouse TGF-β Elisa Kit was purchased from eBioscience (San Diego, CA, USA). Transwell membrane (0.4 μm) was purchased from Corning (NY, USA).

### Cell isolation

CD4^+^ RTEs and CD4^+^ thymic RTE precursors were isolated as previously described^20^. Briefly, CD4^+^ CD8^−^ CD25^−^NK1.1^−^GFP^+^ cells (RTEs) from the liver, lymph nodes or spleen were sorted using FACS Aria II (BD Biosciences). For the purification of CD4^+^ thymic RTE precursors, thymocytes were treated with anti-CD8 mAb and complement to remove CD8^+^ cells. The thymocytes were then stained with a series of antibodies and the cells with the following phenotype CD4^+^ CD8^−^ Qa2^+^ CD69^−^ CD25^−^ CD44^lo^NK1.1^−^ were sorted. The purity of these T cell populations was >97% when analyzed by flow cytometry.

For isolation of non-parenchymal cells (NPCs) in the liver, C57BL/6 mice (CD45.2^+^) were sacrificed and the livers were perfused with 10-20 ml of 0.5 mg/ml collagenase IV in PBS. Livers were mechanically disrupted and incubated with 0.5 mg/ml collagenase IV and 0.25 mg/ml DNase in 20 ml PBS for 30-40 minutes at 37 °C with constant rotation (200 rpm). The resulting cell suspension was then passed through a 150 μm sterile stainless steel meshes. After centrifugation at 500 g for 5 min at 4 °C, the pellet was suspended in an isotonic 35% Percoll solution, and centrifuged at 1000 g for 10 min at room temperature. The resulting pellet was resuspended in ammonium chloride to remove red blood cells and NPCs in the liver were obtained. NPCs were further stained with CD146 and F4/80 to isolate sinusoidal endothelial cells (CD146^+^) and kupffer cells (F4/80^+^).

For isolation of stromal cells in the spleen, spleens from C57BL/6 mice (CD45.2^+^) were mechanically disrupted and incubated with 2 mg/ml collagenase IV and 0.5 mg/ml DNase in 8 ml PBS for 30 minutes at 37 °C with constant rotation (200 rpm). The resulting cell suspension was then passed through a sterile stainless steel meshes and centrifuged at 1500 rpm for 5 min at 4 °C. The cell pellet was resuspended in 4 ml of Ficoll and layered over 4 ml of Ficoll, with 2 ml of PBS further layered on top. After centrifugation at 1700 g for 10 min at 4 °C, stromal cells in the spleen at the interface between PBS and Ficoll were harvested. Flt3 ligand-induced DC (FLDC) was obtained as previously described^21^.

### Low-dose TCR stimulation and TGF-β ELISA

CD4^+^ RTEs were cultured with plate-bound anti-CD3 (0.5 μg/ml) and soluble anti-CD28 (0.25 μg/ml). The supernatants were collected at 72 h of culture and the concentration of TGF-β was measured by Mouse ELISA Kit (eBioscience).

### *In vitro* suppression assay

For iTreg induction, CD4^+^ CD8^−^ CD25^−^ CD62L^+^ CD44^lo^ naive T cells were isolated from C57BL/6 mice (CD45.2^+^) and plated at 2 × 10^5^ cells per well of a 96-well flat-bottom plate with plate-bound anti-CD3 (2 μg/ml), soluble anti-CD28 (1 μg/ml), rhIL-2 (2 ng/ml), rhTGF-β1 (1 ng/ml), anti-IFN-γ (5 μg/ml) and anti-IL-4 (5 μg/ml). The cells were harvested 3 days later and were used in the suppression assay. For the *in vitro* suppression assay, CFSE labeled CD4^+^ CD8^−^ CD25^−^ CD62L^+^ CD44^lo^ naive T cells from CD45.1^+^ C57BL/6 mice were either cultured alone or co-cultured with iTregs, or with CD4^+^ RTEs from the liver or mesenteric lymph nodes of CD45.2^+^ congenic mice at the ratio of 1:1. Anti-CD3 (2 μg/ml) and anti-CD28 (1 μg/ml) were added in the co-culture system. The proliferation of T cells was determined by CFSE dilution of CD45.1^+^ T cells under various culture conditions 2 days later or BrdU incorporation and staining 3 days later.

### T-APC co-culture

CD45.1^+^ CD4^+^ RTE precursors (5 × 10^5^ per well) were stimulated with anti-CD3 (2 μg/ml) and anti-CD28 (1 μg/ml) in the presence of 1 × 10^5^ CD45.2^+^ NPCs or splenic stromal cells, or 7 × 10^4^ LSECs, or KCs, or FLDCs. On the third day, 2 ng/ml of rhIL-2 was added. After 5 days of co-culture, T cells were collected for further analysis of IL-7 responsiveness, FasL and LAG3 expressions by flow cytometry, and IL-10 production by flow cytometry after restimulation with plate-bound anti-CD3 (2 μg/ml) for one additional day. CD45.1^+^ T cells were gated in these analysis. The transwell membrane (0.4 μm) was used to separate LSEC from CD4^+^ RTE precursors when needed. γ-secretase inhibitor (5 μM) was used to block the Notch signaling.

### IL-7 responsiveness

T cells were purified and cultured in RPMI 1640 supplemented with 10% heat-inactivated fetal bovine serum (Hyclone, Logan, UT) with or without 1 ng/ml IL-7. Twenty-four hours later, the cells were collected and stained with anti-CD4, Annexin V, and PI.

### Quantitative RT-PCR

RNA was extracted from the CD4^+^ RTEs from the lymph nodes and liver using TRIZOL (Invitrogen, Grand Island, NY, USA), and cDNA was obtained using the FastQuant RT Kit (TIANGEN, Beijing, China). Quantitative Real-Time PCR was performed using SYBR Green Supermix (Bio-Rad Hemel Hempstead, England, UK) on an iCycler real-time PCR system (Bio-Rad Ltd, Hemel Hempstead, England, UK), with each sample in triplicate. Primers were as follows: Bcl2 forward, 5′-CCATGTGGCTATGCGG-3′; Bcl2 reverse, 5′-ATCAGCCACGCCTAAA-3′; Bcl-xl forward, 5′-GGACCGCGTATCAGAG-3′; Bcl-xl reverse, 5′-GCATTGTTCCCGTAGAG-3′; Bim forward, 5′-CGACAGTCTCAGGAGGAACC-3′; Bim reverse, 5′-CCTTCTCCATACCAGACGGA-3′; Bax forward, 5′-GTGGTTGCCCTCTTCTACTTTG-3′; Bax reverse, 5′-CACAAAGATGGTCACTGTCTGC-3′; Bak forward, 5′-CGAGATGGACAACTTGCCCCTGG-3′; Bak reverse, 5′-CAGCTGATGCCACTCTTAAATAGGCT-3′. The quantifications were based on ΔΔCT calculations and were normalized to GAPDH as loading controls.

### Statistics

The statistical analysis of the results was performed using GraphPad Prism 5 software (San Diego, CA, USA). Unpaired or two-tail paired Student *t*-test was used to evaluate the significance of the differences between two groups. The following terminology is used to denote the statistical significance: *p < 0.05, **p < 0.01, ***p < 0.005.

## Results

### Liver persisting CD4^+^ RTEs have regulatory functions

**Figure 1:**
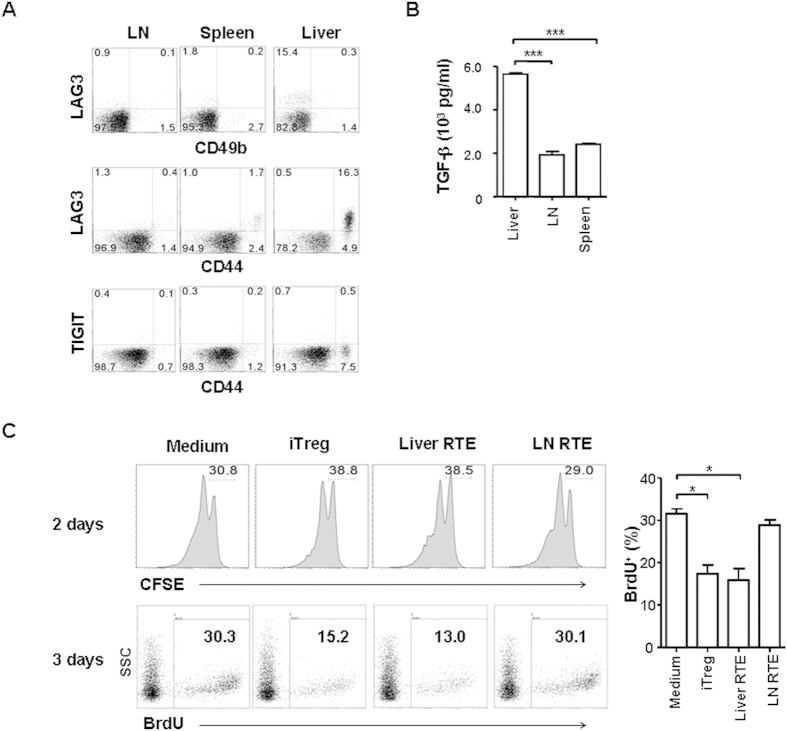
The regulatory functions of liver persisting CD4^+^ RTEs. (**A**) Higher expression of LAG3 by liver CD4^+^ RTEs. GFP^+^ CD4^+^ CD8^−^ CD25^-^NK1.1^-^ RTEs from mesenteric LN, spleen, and liver were examined for LAG3, CD49b, CD44, and TIGIT expression. Three independent experiments were performed and similar results were obtained. (**B**) The production of TGF-β by activated RTEs. GFP^+^ CD4^+^ CD8^−^ CD25^-^NK1.1^-^ RTEs from LN, spleen, and liver were stimulated with plate-bound anti-CD3 (0.5 μg/ml) and soluble anti-CD28 (0.25 μg/ml) for 3 days and the concentration of TGF-β in the supernatant was examined by ELISA. The statistical significance between any two tissues was calculated by Student *t*-test. (**C**) Liver CD4^+^ RTEs suppress the proliferation of naive T cells *in vitro*. CFSE labeled CD4^+^ CD8^−^ CD25^−^ CD62L^+^ CD44^lo^ naive T cells purified from LN of CD45.1^+^ C57BL/6 mice were either cultured alone, or co-cultured with iTregs or CD4^+^ RTEs sorted from the liver or LN of CD45.2^+^ congenic mice at the ratio of 1:1. Anti-CD3 (2 μg/ml) and anti-CD28 (1 μg/ml) were added in the co-culture system. iTregs prepared *in vitro* was used as a positive control. The proliferation of CD45.1^+^ T cells was measured by CFSE dilution 2 days later or BrdU incorporation and staining 3 days later. The numbers in the plots with CFSE labeling indicated the cell ratio that did not enter cell cycle whereas the numbers in the plots with BrdU staining indicated the cell percentages that undergo proliferation. The average percentages of BrdU positive cells (with standard deviation) from three independent experiments were shown on the right and the statistical significance between any two groups was calculated by Student *t*-test.

We previously found that liver persisting CD4^+^ RTEs with CD25^−^ Foxp3^−^ phenotype produced large amounts of IL-10^20^, reminiscent of peripherally derived CD4^+^ Foxp3^-^ type 1 regulatory T cells (Tr1)^22^,^23^,^24^. We thus examined whether liver RTEs express important Tr1 surface markers. About 76.9% GFP^+^ CD4^+^ CD8^−^ CD44^hi^ RTEs in the liver of RAG2p-GFP transgenic mice expressed high level of lymphocyte activation gene (LAG)3 whereas 22.2% and 41.5% CD4^+^ CD44^hi^ RTEs in the LN and spleen, respectively, expressed low level of LAG3 (Fig. 1A). No significant expression of CD49b was found in RTEs (Fig. 1A). The expression of inhibitory receptor TIGIT (T cell immunoglobulin and immunoreceptor tyrosine-based inhibitory motif domain) was also measured and was found negative in RTEs (Fig. 1A). In addition to higher IL-10 production, CD4^+^ RTEs in the liver also secreted more TGF-β than RTEs in the LN and spleen (Fig. 1B).

To assess whether the expression of LAG3, IL-10, and TGF-β in CD4^+^ liver RTEs is associated with suppressor activity indicative of regulatory T cells, CD4^+^ RTEs purified from the liver and LN were co-incubated with anti-CD3- and anti-CD28-activated CD4^+^ CD25^−^ naive T cells from LN. Induced regulatory T cells (Foxp3^+^ iTregs) prepared *in vitro* was used as a positive control. As shown in Fig. 1C, comparable suppression of naïve T cell proliferation, as measured by CFSE on day 2 or BrdU on day 3, was found in the wells co-cultured with liver RTEs and iTregs. LN RTEs had no effect on inhibiting T cell proliferation. These results indicate that some CD4^+^ RTEs acquire regulatory phenotype and function in the liver.

### Liver persisting CD4^+^ RTEs undergo apoptosis

**Figure 2:**
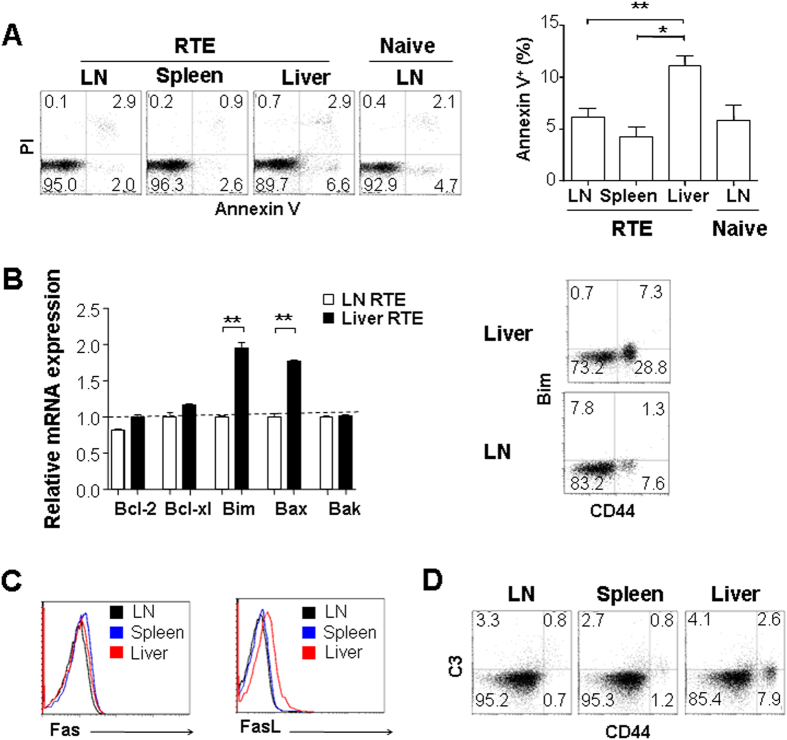
The apoptosis of liver persisting CD4^+^ RTEs. (**A**) RTEs in the liver undergo more apoptosis than those in other lymphoid tissues. GFP^+^ CD4^+^ CD8^−^ CD25^-^ RTEs from mesenteric lymph nodes (LN), spleen, and liver of RAG2p-GFP transgenic mice were stained with Annexin V and Propidium iodide (PI) and analyzed by flow cytometry. The average percentages of annexin V^+^ cells were plotted and the statistical significance between any two tissues was calculated by Student *t*-test. (**B**) CD4^+^ RTEs in the liver express higher level of pro-apoptotic molecules Bim and Bax when compared to RTEs in LN. Total RNA was extracted from CD4^+^ RTEs purified from LNs and liver and quantitative RT-PCR was performed to compare the transcription of Bcl-2, Bcl-xl, Bim, Bax, and Bak (left panel). The comparison of Bim protein level by flow cytometry was also performed in liver and LN RTEs (right panel). (**C**) Expression of FasL, but not Fas in CD4^+^ RTEs is higher in the liver than in LNs and spleen. (**D**) Similar levels of complement C3 deposition on the surface of CD4^+^ RTEs obtained from the liver, LN, and spleen. Two or three independent experiments were performed and similar results were obtained.

We previously found that the survival of liver CD4^+^ RTEs could not be improved by the addition of IL-7^20^. To examine the homeostasis of these RTEs with regulatory phenotype, CD4^+^ RTEs in the liver, LN, and spleen of RAG2p-GFP transgenic mice were compared for their *ex vivo* apoptosis with Annexin V staining. As shown in Fig. 2A, the highest ratio of cell apoptosis was found in liver RTEs. Consistently, liver RTEs expressed about 2-fold higher levels of pro-apoptotic molecules Bim and Bax than LN RTEs (Fig. 2B). The expressions of anti-apoptotic molecules including Bcl-2 and Bcl-xL were similar in RTEs obtained from the liver, LN, and spleen.

Although lowered IL-7 responsiveness in liver RTEs may partially explain the higher death rate of liver RTEs^20^, other death pathways were further examined. Fas/FasL regulates immune homeostasis and tolerance via inducing apoptosis of activated T cells^25^,^26^,^27^,^28^. Compared to CD4^+^ RTEs obtained from LN and spleen, cells from the liver expressed higher level of FasL but not Fas (Fig. 2C).

Hsu, *et al.* found that immature RTEs, particularly NKAP-deficient RTEs, could be eliminated by the classical complement activation pathway^29^. We thus examined whether more complement C3 was bound to liver RTEs. As shown in Fig. 2D, similar levels of C3 were found on the surface of RTEs obtained from the liver, LN, and spleen, suggesting that complement-mediated cell deletion may not be specifically involved in the regulation of RTEs in the liver. Together, these results suggest that Fas/FasL pathway and Bim-mediated mitochondria pathway may together lead to the increased apoptosis of autoreactive CD4^+^ RTEs in the liver.

### LSECs induce the tolerogenic phenotype of CD4^+^ RTEs

**Figure 3:**
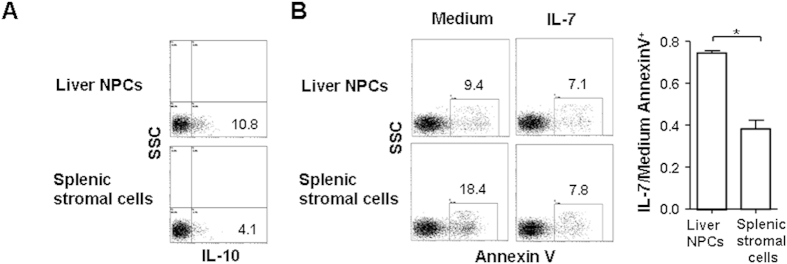
Liver NPCs induce the tolerogenic phenotype in CD4^+^ RTEs. CD4^+^ RTE precursors (CD4^+^ CD8^−^ Qa2^+^ CD69^−^ CD44^lo^ thymocytes, 5 × 10^5^ cells per well) were stimulated with anti-CD3 (2 μg/ml) and anti-CD28 (1 μg/ml) in the presence of 1 × 10^5^ NPCs or 1 × 10^5^ splenic stromal cells. On the third day, 2 ng/ml rhIL-2 was added. After 5 days of co-culture, the T cells were collected for further analysis. (**A**) Increased IL-10 production in T cells co-cultured with liver NPCs. T cells were re-stimulated with plate-bound anti-CD3 (2 μg/ml) for one day and IL-10 production was measured by flow cytometry. (**B**) Reduced IL-7 responsiveness in T cells co-cultured with liver NPCs. T cells were cultured in the presence or absence of 1 ng/ml IL-7 for one day and Annexin V staining was used to measure the apoptosis of T cells. The apoptosis of T cells cultured in the presence of IL-7 was calculated against that of cells cultured in medium and the mean ratios of three independent experiments were shown on the right.

Using *Ltbr*^−/−^ mice with defects in secondary lymphoid organ, we previously found that the retention and activation of a proportion of CD4^+^ RTEs mainly occurred in the liver, independent of the signals from LN and spleen. This suggests that CD4^+^ RTEs’ acquisition of tolerogenic phenotype depends on liver microenvironment^20^. To investigate the tolerogenic role of cellular components in the liver, an *in vitro* T-APC co-culture system was applied and the mixed population of liver stromal cells was first tested. Stromal cells purified from the spleen were used as controls. As RTEs from LN, spleen, and liver showed different properties that may be acquired during their presence in these organs, we purified thymic CD4^+^ RTE precursors (Qa2^+^ CD69^−^ CD44^lo^ CD25^−^ CD4^+^ CD8^−^ mature SP thymocytes) for the T-APC co-culture^20^. The IL-7 responsiveness, the expression of IL-10, FasL, and LAG3 in T cells 4 days after co-culture were examined. Undetectable level of FasL and LAG3 expression in CD4^+^ RTE precursors was found before the *in vitro* co-culture (data not shown). Compared to splenic stromal cells, T cells co-cultured with liver non-parenchymal cells (NPCs) showed nearly 2.5-fold more IL-10 production (Fig. 3A). The survival of T cells co-cultured with liver NPCs was better than that of T cells with splenic stromal cells (Fig. 3B). However, the addition of IL-7 reduced the apoptosis of T cells by half in the wells co-cultured with splenic stromal cells whereas it did not decrease the apoptosis of T cells co-cultured with liver NPCs (Fig. 3B). These results indicate the important roles of liver NPCs in inducing IL-10 expression and reducing IL-7 responsiveness in RTEs.

**Figure 4:**
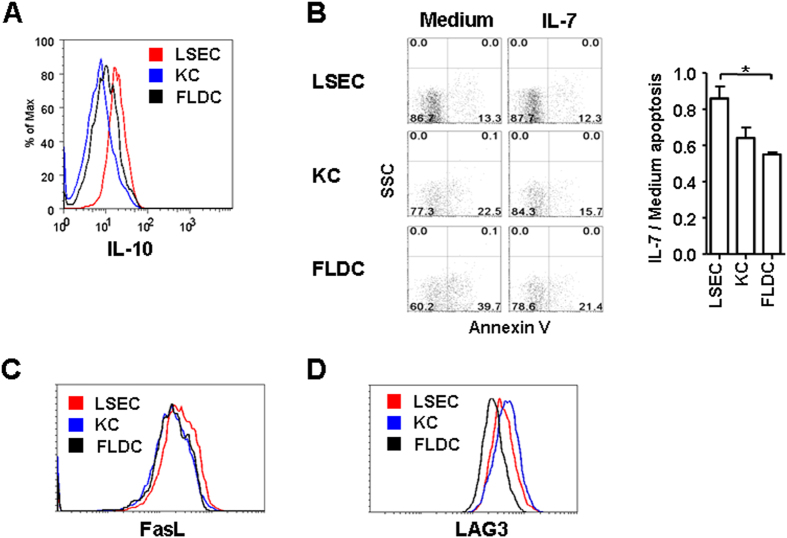
LSECs contribute to the tolerogenic induction of CD4^+^ RTEs. CD4^+^ RTE precursors (5 × 10^5^ per well) were stimulated with anti-CD3 (2 μg/ml) and anti-CD28 (1 μg/ml) in the presence of 7 × 10^4^ LSECs, KCs or FLDCs. On the third day, 2 ng/ml of rhIL-2 was added. After 5 days of co-culture, T cells were collected for further analysis. (**A**) Enhanced IL-10 production in T cells co-cultured with LSECs. T cells were restimulated with plate-bound anti-CD3 (2 μg/ml) for one day and IL-10 production by T cells was measured by flow cytometry. (**B**) Reduced IL-7 responsiveness in T cells co-cultured with LSECs. The apoptosis of T cells cultured in the presence of IL-7 was calculated against that of cells cultured in medium and the mean ratios of three independent experiments were shown on the right. (**C**) Increased expression of FasL in T cells co-cultured with LSECs. (**D**) Increased expression of LAG3 in T cells co-cultured with LSECs and KCs. Data are representative of two independent experiments.

LSECs and KCs are two main stromal cell types involved in the liver tolerogenic regulation. To investigate their roles on RTE tolerance, thymic RTE precursors were co-cultured with purified liver LSECs and KCs. The co-culture with Flt3 ligand induced DCs (FLDCs) was used as a control. As shown in Fig. 4A, CD4^+^ RTE precursors co-cultured with LSECs revealed highest level of IL-10 production whereas those cultured with KCs produced lowest IL-10. In the absence of IL-7, LSECs showed highest capability in improving the survival of RTE precursors (Fig. 4B). In the presence of IL-7, however, the survival of T cells co-cultured with KCs and FLDCs was greatly improved whereas that of T cells with LSECs was not. After 4-day culture with LSECs, RTE precursors expressed high level of FasL (Fig. 4C). No significant FasL up-regulation was found in T cells co-cultured with KCs and FLDCs. Both KCs and LSECs induced the expression of suppressor marker LAG3 in RTE precursors whereas FLDCs did not (Fig. 4D). These results suggest that LSECs may be the main contributors in the acquisition of tolerogenic phenotype in autoreactive CD4^+^ RTEs and KCs may also participate in the induction of suppressor function.

### Cellular interaction is required for and Notch signaling pathway is partially involved in the tolerance induction of CD4^+^ RTEs by LSECs

**Figure 5:**
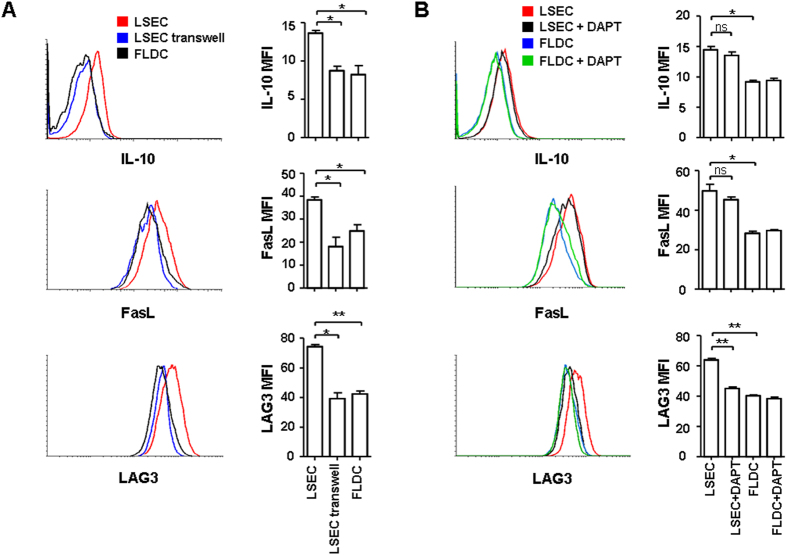
Cellular interaction between LSECs and T cells and Notch signaling are important in the tolerance induction of CD4^+^ RTEs. (**A**) Cellular interaction is required for the tolerance induction of CD4^+^ RTEs by LSECs. CD4^+^ RTE precursors were either cultured with LSECs in the same well, or in the upper well of the transwell system, with LSECs seeded on the bottom of the well. The T cell culture with FLDCs in the same well was used as a control. Anti-CD3 (2 μg/ml) and anti-CD28 (1 μg/ml) were added in the co-culture system. On the third day, 2 ng/ml of rhIL-2 was added. After 5 days of co-culture, T cells were collected and restimulated with plate-bound anti-CD3 (2 μg/ml) for one additional day and IL-10 expression in T cells was measured by flow cytometry. T cells were also examined for their expressions of FasL and LAG3. The mean fluorescence intensities (and standard deviation) of IL-10, FasL, and LAG3 in three experiments were shown on the right panel. (**B**) The Notch signaling pathway contributes to CD4^+^ RTEs’ tolerance induction by LSECs. CD4^+^ RTE precursors were cultured with LSECs or FLDCs in the same well in the presence of anti-CD3 (2 μg/ml) and anti-CD28 (1 μg/ml). DAPT, a γ-secretase inhibitor (5 μM) was added in the co-culture system to block the Notch signaling. On the third day, 2 ng/ml of rhIL-2 was added. After 5 days of co-culture, CD4^+^ RTE precursors were collected and analyzed for the expressions of IL-10, FasL, and LAG3. The mean fluorescence intensities (and standard deviation) of IL-10, FasL, and LAG3 in three experiments were shown on the right panel.

To investigate whether cellular interaction is required for the induction of CD4^+^ RTE tolerance by LSECs in the liver, we compared the phenotype of RTE precursors cultured with LSECs in the same well or separately in the transwell system. As shown in Fig. 5A, the T cells’ up-regulation of IL-10, FasL, and LAG3 was only found in the wells having CD4^+^ RTE precursors and LSECs cultured together. This suggests that direct cellular interaction with LSECs is required for the induction of tolerogenic phenotype of CD4^+^ RTEs.

Recently, the Notch signaling pathway activated by hepatocytes or LSECs has been reported to induce IL-10 production in Th1 cells^30^,^31^. We thus examined whether the phenotype of CD4^+^ RTE precursors acquired from T-LSEC co-culture depends on Notch. As shown in Fig. 5B, the addition of γ-secretase inhibitor (DAPT) which could block Notch activation prevented LAG3 upregulation in T cells. The induction of IL-10 and FasL expression, however, was not affected. These results suggest that the Notch pathway partially contributes to the liver tolerance of CD4^+^ RTEs.

## Discussions

We have previously found a unique population of CD4^+^ RTEs in the liver that could induce severe inflammation and T cell infiltration in the lung and colon following transfer into RAG^−/−^ recipients^20^. These autoreactive CD4^+^ liver RTEs have activated phenotype (CD44^hi^CD62L^lo^) and more than 40% of CD44^hi^CD4^+^ RTEs expressed CXCR3. The majority of these cells is Foxp3^−^ and does not express inhibitory molecules PD-1, CD73, and FR4. The acquirement of this unique phenotype is independent of secondary lymphoid organs and relies on RTEs entering the liver. Upon low concentration of anti-CD3 and anti-CD28 stimulation, CD4^+^ RTEs in the liver proliferated faster and produced higher levels of IL-10 as well as IL-2, TNF-α, IFN-γ, and IL-4 than RTEs in the lymph nodes and spleen^20^. The current study further found high expression of LAG3, FasL, and TGF-β but not CD49b in CD4^+^ liver RTEs. Compared to iTregs, these liver RTEs showed similar level of inhibition of naïve T cell proliferation. Thus, the regulatory features of CD44^hi^Foxp3^−^ CD4^+^ RTEs acquired in the liver under steady state condition are distinct from those of the conventional CD4^+^ Foxp3^+^ Treg cells, CD4^+^ Foxp3^-^LAG3^+^ CD49b^+^ Tr1 cells, and IL-10-producing T helper 1 (Th1) cells^22^,^23^,^24^,^31^,^32^,^33^,^34^.

The modulation of immune responses in the liver, in particular, the induction of tolerance is mediated by specialized liver-resident APCs, including LSECs, KCs, tolerogenic DCs, and hepatocytes^35^,^36^,^37^. Among them, LSECs have been shown to promote the anergy of CD8^+^ T cells, the differentiation of Foxp3^+^ CD4^+^ or Foxp3^−^CD4^+^ regulatory T cells, and the differentiation of IL-10^+^ IFN-γ^+^ Th1 cells 11,12,13,14,15,38. In our results, LSECs were more efficient than KCs in inducing IL-10 and FasL expressions and down-regulating IL-7 responsiveness in CD4^+^ RTEs. LSECs could also induce LAG3 expression in CD4^+^ RTEs, agreeing well with the findings that LSECs induce the differentiation of LAG3^+^ Foxp3^−^ Tr1 cells^14^,^39^.

PD-1-PD-L1 interaction, LSECtin-CD44 interaction, membrane-bound TGF-β, and Notch pathway have been reported to contribute to LSEC-induced T cell tolerance^11^,^13^,^40^,^41^,^42^,^43^,^44^. As liver RTEs are PD-1^lo^ IL-2^+^ IFN-γ^+^, PD-L1 and LSECtin may not play a major role in RTEs’ tolerance induction. It has been found that LSECs could express all four Notch ligands, Jagged1, Jagged2, Dll1, and Dll4^15^,^40^. The interaction of Notch with its ligands induced IL-10 production in activated T cells or differentiated Th1 cells or PD-1 expression in activated T cells, resulting in the suppression of immune responses or maintenance of T cell exhaustion^15^,^40^,^45^,^46^. Interestingly, our results with γ-secretase inhibitor suggest for the first time that the activation of Notch pathway is involved in LSECs’ capability of LAG3 induction in CD4^+^ RTEs. Whether membrane-bound TGF-β is involved and coordinates with the Notch pathway to induce other tolerogenic phenotype in RTEs awaits further investigation. Together, the fenestrated LSECs that line the hepatic sinusoids may provide antigen-specific and Notch signals and likely other signals during transmigration of CD4^+^ RTEs across the endothelium, and induce the responding RTEs to become tolerogenic and express regulatory functions. KCs or even other liver APCs may be also involved in this tolerogenic regulation.

The regulatory functions of CD4^+^ liver RTEs to other T cells or even APCs are probably via IL-10 and/or FasL expression. IL-10 may activate STAT3 and its target gene expression in other T cells or even autoreactive CD4^+^ RTEs themselves and eventually limiting the activation and inflammatory cytokine production of these T cells^47^,^48^,^49^. FasL expression in CD4^+^ RTEs may facilitate the binding to its receptor Fas/CD95 on T cells and induce extrinsic pathway of cell apoptosis. The interaction of FasL and Fas leads to the recruitement of Fas-associated protein with death domain (FADD) and the activation of caspase 8. Activated caspase 8 can either directly cleave pro-caspase-3 and -7, or cleave the BH3-only protein Bid to activate Bax/Bak-mediated mitochondrial apoptotic pathway^50^. Whether high expression of IFN-γ by CD4^+^ liver RTEs plays an important regulatory role remains elusive. However, IFN-γ was reported to contribute to antigen-specific regulatory cell differentiation, development, or clonal deletion, thus maintaining immune tolerance 51,52.

Notably, the impact of the regulatory function of CD44^hi^CD4^+^ liver RTEs may be limited under steady state condition as these cells expressed high levels of FasL and Bim, responded poorly to IL-7-mediated cell survival, an indication of deletion of these CD4^+^ RTEs in the liver. Indeed, after being transferred into lymphoreplete mice via tail vein injection, the number of donor thymic CD4^+^ RTE precursors in the liver was decreased within 2-4 weeks^20^, suggesting that apoptotic cell death is the final fate of CD44^hi^CD4^+^ RTEs in the liver after a transient activation and tolerance induction. However, current results do not exclude the possibility that under special conditions these CD4^+^ CD44^hi^ LAG3^+^ FasL^+^ IL-10^+^ IFN-γ^+^ RTEs in the liver may participate in the regulation of immune responses^20^,^53^.

Taken together, our data reveal that some newly generated CD4^+^ thymic emigrants acquire tolerogenic and suppressor phenotype in the liver and eventually undergo apoptosis. LSECs and the activation of Notch signaling pathway contribute to RTEs’ tolerance induction.

## Additional Information

**How to cite this article**: Xu, X. *et al.* Liver sinusoidal endothelial cells induce tolerance of autoreactive CD4^+^ recent thymic emigrants. *Sci. Rep.* **6**, 19861; doi: 10.1038/srep19861 (2016).
